# Oxytocin receptor gene, childhood maltreatment and borderline personality disorder features among male inmates in China

**DOI:** 10.1186/s12888-020-02710-0

**Published:** 2020-06-24

**Authors:** Min Zhang, Na Liu, Haocheng Chen, Ning Zhang

**Affiliations:** 1 School of Psychology, Nanjing Normal University, Nanjing, 210000 China; 2 Medical Psychology Department, Nanjing Brain Hospital Affiliated to Nanjing Medical University, Nanjing, 210000 China; 3 The Forth School of Clinical Medicine, Nanjing Medical University, Nanjing, 210000 China

**Keywords:** Borderline personality disorder, OXTR, Single-nucleotide polymorphism, Childhood maltreatment

## Abstract

**Background:**

Borderline personality disorder (BPD) is caused by a variety of biological and environmental factors. Accumulating evidence suggests that childhood maltreatment is a risk environmental factor in the development of BPD, but research on the genetic pathology of BPD is still in its early stages, and very little is known about the oxytocin receptor (OXTR) gene. The purpose of this study is to further explore the interactive effects between OXTR gene polymorphisms and childhood maltreatment on BPD risk.

**Methods:**

Among the 1804 Chinese Han male inmates, 765 inmates who had BPD or antisocial personality disorder (ASPD) or highly impulsive or violent crime were considered as high-risk inmates and included in this study. Childhood maltreatment, BPD, antisocial personality disorder (ASPD) and impulsivity were measured by self-reported questionnaires. Peripheral venous blood was collected for the genotype test.

**Results:**

Analyses revealed that the BP group (inmates with BPD features) had higher rs53576 AA genotype frequency and rs237987 AA genotype frequency than the non-BP group, while the statistical significances were lost after Bonferroni correction. Total childhood maltreatment score, emotional abuse and neglect could positively predict BPD risk. Among the high-risk samples, rs53576 GG genotype carriers had higher BPD scores at higher levels of physical abuse and sexual abuse and had lower BPD scores at lower levels of physical abuse and sexual abuse.

**Conclusions:**

The findings suggest that the interaction between OXTR gene variations and childhood maltreatment is an important mechanism for the development of BPD. The moderating role of the OXTR gene provides evidence for gene plasticity.

## Background

Borderline personality disorder (BPD) is characterized by a pervasive pattern of emotional instability, interpersonal tension, high impulsiveness, identity disorder and cognitive impairment [1]. BPD has been estimated to have 10% mortality caused by suicide [2]. Therefore, BPD imposes enormous economic and psychological burdens, highlighting the necessity of elucidating the pathological mechanism of BPD. Decades of research demonstrate that BPD is caused by a variety of biological and environmental factors, and the interaction between these two factors has been emphasized recently [3].

Twin studies suggested that 35–67% of the variance in BPD risk is attributable to genetic factors [4, 5]. Great efforts have been made to elucidate the possible genetic background of BPD, and the current polymorphism studies mainly focused on the serotonin receptor (HTR), tryptophan hydroxylase (TPH), monoamine oxidase A (MAOA), catechol-O-methyltransferase (COMT) and dopamine transporter (DAT) genes [6–9]. However, very few studies have investigated the association between oxytocin receptor (OXTR) gene and BPD.

Oxytocin is a peptide hormone synthesized in the paraventricular and the supraoptic nucleus of the hypothalamus. Oxytocinergic neurotransmission is mediated by the oxytocin receptors. The OXTR gene located on chromosome 3p25 is associated with encoding and activity of oxytocin receptors [10]. Several single nucleotide polymorphisms (SNPs) in the OXTR gene have been proven to be related to social cognition and behavior as well as greater susceptibility to psychiatric disorders. The OXTR SNPs rs53576 and rs2254298 were frequently studied in human [11, 12]. Specifically, previous studies found that OXTR rs53576 was associated with prosocial behavior including positive parenting [13] and better empathic ability [14], as well as psychiatry disorders such as schizophrenia [15] and depression [16]. Similar to the SNP rs53576, numerous studies have demonstrated that the SNP rs2254298 was implicated in infant attachment security [17], higher social cognition [18], depression [19], autism spectrum disorder (ASD) [20]. However, the associations between the two SNPs and BPD remain to be explored.

Although other OXTR SNPs were relatively less studied, evidence has accumulated suggesting that OXTR gene polymorphisms are associated with the susceptibility to psychopathology. For instance, a previous study found that the haplotype ATTGA (rs237897-rs13316193-rs237889-rs2254298-rs2268494) was associated with ASD [21]. In addition, six SNPs in the OXTR gene including rs1042778, rs2268490, rs13316193, rs2254298, rs237889 and rs13311693 were linked to callous-unemotional (CU) traits [22]. Malik et al. found that rs6770632 and rs1042778 might be associated with aggressive behaviors in females and males, respectively [23]. In the present study, we focused on the eight SNPs in the OXTR gene (rs237897, rs13316193, rs237889, rs2254298, rs2268494, rs1042778, rs53576 and rs6770632), in order to explore the relationship between OXTR genetic variants and BPD.

As mentioned above, OXTR SNPs not only affect the prosocial behavior of individuals, but also affect the development of psychiatry disorders. The contradictory effect may be attributable to environmental factors, which means the interaction between the gene and the environment (G × E) may play a role in the development of BPD. The function of OXTR gene may vary according to social stimulus which conformed to the social sensitivity hypothesis [24, 25]. Specifically, OXTR gene is related to increased sensitivity to the surroundings, both in favorable or negative contexts. A recent study confirmed this hypothesis that individual who carried OXTR rs53576 A-allele had higher levels of BPD symptoms under negative family conditions and lower levels under positive conditions [26]. Considering that the function of OXTR is environment-based, it is necessary to explore the environmental factors of BPD.

Regarding the influence of environmental factors on BPD, previous studies had investigated the associations between family environment [27], childhood maltreatment [28], early life stress [29] and BPD. In the present study, we sought to shed more light on childhood maltreatment and its interaction with OXTR gene. Several lines of evidence show that there are interactions between the OXTR gene polymorphisms and childhood maltreatment; for instance, OXTR rs53576 G-allele carriers who had experienced maltreatment in childhood were observed to increase conduct problems in adolescence [30], and maltreated individual who carried the rs237885 TT genotype in OXTR gene had an increased risk of aggression [31]. In addition, the interaction between the OXTR gene and childhood maltreatment could affect the function or structure of the brain, especially the amygdala [32], which is responsible for emotional regulation what BPD patients lack. However, research on the interaction between the OXTR gene and childhood maltreatment for the development of BPD is very scarce [26, 33].

Reviewing researches on the interaction between OXTR gene and childhood maltreatment, we found that rs53576 has emerged as an important SNP in the understanding of the G × E interactions. Previous studies provide evidence that the GG genotype carriers are assumed to show higher trust-related behaviors and empathic concern in comparison to A allele carriers [34, 35]. However, in face of adversity such as childhood abuse, the GG genotype carriers may be more vulnerable to be affected by negative early-life experiences. For instance, Bradley et al. found that the GG genotype carriers exposed to childhood maltreatment exhibited emotional dysregulation and disorganized attachments styles [36]. Hostinar et al. showed that GG carriers perceived significantly lower social support and reported higher levels of internalizing symptoms than A-carriers among maltreated adolescents [37].

Previous study suggested that most inmates had a personality disorder when they committed the crime. BPD and antisocial personality disorder (ASPD) are the most common personality disorders in prison setting [38]. Several studies have provided strong evidence that ASPD and BPD are risk factors of violence [39–41], and the association between the personality disorders and violence may be a result of impulsiveness [42]. Therefore, individuals with BPD and ASPD features, violent criminals and high impulsive prisoners are all high-risk groups in prison, which may lead to a great deal of hardship for prison management, especially among male inmates. Although the prevalence of BPD in women is higher than that in men [43], the crime rate of men is higher than that of women [44]. Considering the absence of such study on this group in China, male high-risk inmates were chosen as our study subjects.

To explore the role of the OXTR gene in contributing risk to BPD features, and whether these polymorphisms which may be related to the variations of OXTR gene have different effects on BPD features depends on the levels of childhood maltreatment in male high-risk inmates, we sought to test three hypotheses: (1) participants with borderline personality features would show differences in OXTR genotype frequency compared with those without borderline personality features; (2) childhood maltreatment and its dimensions could positively predict BPD among male high-risk inmates in China; and (3) the OXTR gene polymorphisms would interact with childhood maltreatment or its dimensions to predict BPD risk.

## Methods

### Participants

A total of 1804 male inmates from an adult male prison in Jiangsu Province, China initially participated in the present study, and all participants were Chinese Han individuals. The study was approved by the Institutional Review Board of Nanjing Brain Hospital, and a consent form for this study was prepared. We carried out the study following the principle of the Helsinki Declaration. For the compensation for the participants, some lectures, psychoeducation about mental health issues for inmates and some didactic lessons and supervise program about mental illness for prison clinical faculty and staff were regularly arranged for their support of the research. After obtaining informed consent to conduct the self-report survey, all of them were investigated by a structure questionnaire asking about sociodemographic characteristics, personality disorders, history of childhood maltreatment and impulsivity. Considering that the present study aimed to investigate the high-risk population in prison, participants who met one of the following conditions would be included: (1) BPD or ASPD score in the Personality Diagnostic Questionnaire-4 (PDQ-4) ≥ 4 [45]; (2) Barratt Impulsiveness Scale-11 (BIS-11) score ≥ 62 [46]; (3) history of violent crime, such as intentional homicide, intentional injury, and robbery. Among the 1804 participants, 1050 (58.2%) inmates were considered as high-risk population. 765 (72.86%) valid blood samples were obtained from the 1050 inmates after obtaining informed consent for blood collection. Finally, 765 subjects with questionnaire data and blood samples were included in the study. Those who had previously been diagnosed with chronic heart, liver and kidney diseases; had a history of nervous system diseases or mental disorders; or had a long history of medication use were excluded.

Among the 765 inmates with high risk, 342 (44.71%) inmates met the criteria for borderline personality features (BP group), while 423 (55.29%) inmates did not meet the criteria (non-BP group), i.e., a score of 4 points on the BPD subscale of the PDQ-4. The average age of the high-risk subjects was 33.50 years old (SD = 9.01). Most subjects had a low level of education, of which 33.7% (*n* = 258) had attended school for less than or equal to 6 years, 62.2% (*n* = 476) for 7–12 years, and the remaining 4.1% (*n* = 31) for 13 years or more. Nearly half of the subjects had relatively stable partner relationships, as 46.4% (*n* = 355) of them were in married or cohabiting, 38.7% (*n* = 296) of them were unmarried and the remaining 14.9% (*n* = 114) were divorced or widowed. Among the high-risk sample, 57.6% (*n* = 441) had children.

### Measures

#### Personality diagnostic Questionnaire-4 (PDQ-4)

The PDQ-4 was administered to assess BPD and ASPD which is a further revision to PDQ based on DSM-IV [45]. This self-reported questionnaire consisted of 85 items, which are used to assess 10 types of personality disorders (paranoid, schizophrenic, split, hysteric, narcissistic, borderline, antisocial, avoidant, dependent and compulsive). In this study, the BPD subscale (9 items) of the PDQ was used to assess the borderline personality traits of the subjects instead of clinical diagnosis, with higher scores reflecting correspondingly higher levels of BPD. The ASPD subscale (8 items) was used to screen whether the inmates had the risk of ASPD. PDQ-4 demonstrated good psychometric properties when applied to clinical samples and general population [47, 48]. The internal consistency as reflected by Cronbach’s alpha for the 85 items in the present study was 0.919, and the internal consistency for BPD and ASPD subscales was 0.689 and 0.675, respectively.

#### Childhood trauma questionnaire short form (CTQ-SF)

The CTQ-SF, compiled by Bernstein DP and Flink L [49], was administered to assess five categories of childhood abuse and neglect including emotional abuse, physical abuse, sexual abuse, emotional neglect and physical neglect. This instrument is a 28-item retrospective self-reported questionnaire, and the items follow a Likert-type response style from 1 to 5, which is organized to reflect the frequency of abusive experiences (never true, rarely true, sometimes true, often true, very often true). Each dimension has 5 items, and the remaining 3 items are validity evaluation, so each dimension is scored between 5 ~ 25 and the total score is 25 ~ 125. The CTQ and its dimensions (other than physical neglect) had good reliability and validity in Chinese population [50]. The internal consistency as reflected by Cronbach’s alpha for the 28 items in the present study was 0.897, and the internal consistency for each subscale was 0.801 (emotional abuse), 0.875 (physical abuse), 0.878 (sexual abuse), 0.843 (emotional neglect), and 0.628 (physical neglect), respectively.

#### Barratt impulsiveness Scale-11 (BIS-11)

The BIS-11 was administered to assess impulsivity which was originally developed by Barratt in 1959. This self-reported questionnaire consists of 26 items, which is answered on a 4-point Likert scale ranging from 1 to 4 (rarely, occasionally, often, almost always). Three types of impulsivity, including attentional impulsiveness, motor impulsiveness, and non-planning impulsiveness, are measured, with the higher scores of each dimension reflecting corresponding higher levels of such impulsiveness. In this study, the total score of BIS-11 was used to screen impulsivity of subjects in prison. According to the previous studies in Chinese population, the BIS-11 and its dimensions demonstrated good reliability and validity [46]. The internal consistency as reflected by Cronbach’s alpha for the 26 items in the present study was 0.743.

### SNPs selection, DNA extraction and genotyping

According to the previous studies [11–23], the SNPs which were found to be related to the development of psychiatric disorders were selected from the 1000 genome database of Han Chinese in Beijing, China (CHB) (http://www.internationalgenome.org/). We focused on eight SNPs in the OXTR gene, including rs1042778, rs13316193, rs2254298, rs2268494, rs237889, rs237897, rs53576, rs6770632.

Venous blood samples were collected from 765 subjects (342 subjects in BP group and 423 subjects in non-BP group). Genetic DNA was extracted using Sunshinebio TM blood genomic DNA Extraction Kit (Sunshinebio, Nanjing, China) following the manufacturer’s instructions. The extracted DNA was diluted to a concentration of 50 ng/μL. Genotyping of the OXTR SNPs applied multiple PCR technology and high-throughput sequencing technology. The primers showed in Additional file 1: Table S1. Multiple PCR amplification was performed using PERKIN ELMER Gene Amp PCR system 9600 (Applied Biosystems, Shanghai, China). Different samples were distinguished by different Barcode primers, and the high flux sequencing of the amplified products was performed using ABI PRISM 377 DNA Sequencer (Applied Biosystem, Shanghai, China). Several samples failed to be genotyped, but the call rates for all SNPs almost reached 95% (Table 2).

### Statistical analysis

Statistical analyses were performed using Statistical Product and Service Solutions (SPSS) 22.0. Linkage disequilibrium (LD) and haplotype construction were performed using Haploview 4.1 software. First, descriptive statistics were used to present the demographic characteristics of the subjects and the distribution of alleles of all investigated SNPs. Second, the Hardy-Weinberg equilibrium (HWE) for genotypes was assessed by a chi-squared goodness-of-fit test to examine whether the sample was a genetic balance population. Third, the differences in demographic characteristics and genotypes between the BP group and the non-BP group were examined by performing a series of t-tests and chi-squared goodness-of-fit tests. Fourth, one-way analysis of variance (ANOVA) and chi-squared goodness-of-fit tests were used to test for differences in demographic characteristics stratified by genotypes. Fifth, association analysis between childhood maltreatment subtypes and BPD level was performed with a multiple linear regression. Lastly, the interactions between OXTR gene polymorphisms (rs53565: GG/AG + AA; rs237897: GG/AG + AA) and childhood maltreatment were analyzed using hierarchical linear regressions.

All reported *p* values were two-sided. The nominal significance threshold was set at 0.05 and Bonferroni corrected *p*-value threshold for multiple testing was set at 0.00625 (0.05/8 SNPs) or 0.0125 (0.05/4 haplotypes).

## Results

### Subjects characteristics

According to the BPD scores, the high-risk subjects were divided into a BP group and a non-BP group, 342 (44.71%) cases and 423 (55.29%) cases respectively. As presented in Table 1, groups were similar in age, education, marital status, and reproductive status (*p* > 0.05). Subjects in the BP group obtained higher scores on the overall CTQ as well as its five subtypes than those in the non-BP group (*p* < 0.001).

**Table 1:** Demographic characteristics of subjects

Characteristic	BP group (*n* = 342)	Non-BP group (*n* = 423)	Statistic	*p*
Age M ± SD	33.86 ± 9.03	33.22 ± 8.81	0.979^a^	0.328
Education, *n*(%)				
Less than or equal to 6 years	120 (35.1)	138 (32.6)		
7–12 years	211 (61.7)	265 (62.6)		
More than or equal to 13 years	11 (3.2)	20 (4.7)	1.434^b^	0.488
Marital status, *n*(%)				
unmarried	130 (38.0)	166 (39.2)		
Married/cohabitation	157 (45.9)	198 (46.8)		
Divorce/widowed	55 (16.1)	59 (13.9)	0.685^b^	0.710
Reproductive status				
Nonbirth	140 (40.9)	184 (43.5)		
Birth	202 (59.1)	239 (56.5)	0.509^b^	0.476
CTQ total score M ± SD	51.79 ± 17.53	42.92 ± 15.55	7.320^a^	< 0.001
Emotional abuse	9.26 ± 4.05	7.73 ± 3.59	5.460^a^	< 0.001
Physical abuse	9.06 ± 4.63	7.48 ± 3.75	5.090^a^	< 0.001
Sexual abuse	8.24 ± 4.08	6.83 ± 3.58	5.029^a^	< 0.001
Emotional neglect	14.35 ± 5.83	11.73 ± 5.34	6.413^a^	< 0.001
Physical neglect	10.87 ± 3.83	9.13 ± 3.78	6.274^a^	< 0.001

Note: ^a^ Independent-sample t-test, ^b^ chi-squared goodness-of-fit test;

*CTQ* Childhood Trauma Questionnaire

### Association analysis between OXTR SNPs and BP

Table 2 presents the basic information about the eight OXTR SNPs which were genotyped in the present study. The minor allele frequencies (MAF) of these eight SNPs were similar to those in the 1000 genome database of CHB. The genotypes in the high-risk inmates conformed to Hardy-Weinberg equilibrium (HWE) (*p* > 0.05). HWE tests also conducted in the subgroups. The results indicated that, except OXTR rs1042778 in the non-BP group (*χ*^*2*^ = 10.396, *p* = 0.001), all other SNPs conformed to HWE in both groups (*χ*^*2*^ = 0.019–2.197, *p* > 0.05). Considering the SNP rs1042778 did not deviate from HWE in the whole sample of high-risk inmates (Table 2), such deviation in the non-BP group may be due to the reduction of sample size.

**Table 2:** Basic information of the SNPs in the current study

rs ID	Position	Alleles	Minor allele in Asian	MAF in High-risk inmates	MAF in CHB	Call rate (100%)	*p* for HWE
rs237897	3: 8808285	A/G	G	0.263	0.252	0.949	0.667
rs13316193	3: 8802743	T/C	C	0.155	0.189	0.984	0.161
rs237889	3: 8802483	T/C	T	0.415	0.413	0.983	0.325
rs2254298	3: 8802228	A/G	A	0.318	0.325	0.984	0.228
rs2268494	3: 8802046	A/T	A	0.075	0.117	0.989	0.712
rs1042778	3: 8794545	T/G	T	0.083	0.073	0.975	0.064
rs53576	3: 8804371	A/G	G	0.283	0.306	0.966	0.841
rs6770632	3: 8793724	A/C	A	0.005	0.01	0.988	0.884

Note: *MAF* minor allele frequency, *CHB* Han Chinese in Beijing, China, *HWE* Hardy-Weinberg equilibrium

As showed in Table 3, the distributions of age showed significant differences in the genotype frequencies of rs2268494 (*p* < 0.007). Chi-squared tests showed that there were significant differences in the genotype frequencies of rs237889 among different levels of education (*p* < 0.05). In addition, significant differences in the genotype frequencies of rs53576 were observed among different marital status (*p* < 0.05). However, after Bonferroni correction, only the relationship between age and rs2268494 remained statistically significant. Apart from the results mentioned above, age (*F* = 0.047–1.134, *p* > 0.05), education (*χ*^*2*^ = 0.728–7.264, *p* > 0.05), marital status (*χ*^*2*^ = 3.605–8.685, *p* > 0.05), and reproductive status (*χ*^*2*^ = 0.081–5.495, *p* > 0.05) were not found to be statistically significant in the genotype frequencies of other SNPs.

**Table 3:** Demographic characteristics stratified by genotype

Characteristic	Genotype			Statistic	*p*
rs2268494	AA	AT	TT		
Age M ± SD	24.20 ± 4.43	35.97 ± 10.72	33.13 ± 8.49	7.429^a^	0.001
Education, n(%)					
rs237889	TT	TC	CC		
Less than or equal to 6 years	45 (33.1)	117 (33.2)	89 (33.7)		
7–12 years	87 (64.0)	211 (59.9)	172 (65.2)		
More than or equal to 13 years	4 (2.9)	24 (6.8)	3 (1.1)	13.102^b^	0.011
Marital status, n(%)					
rs53576	AA	AG	GG		
unmarried	162 (42.7)	105 (34.8)	22 (37.9)		
Married/cohabitation	166 (43.8)	156 (51.7)	21 (36.2)		
Divorce/widowed	51 (13.5)	41 (13.6)	15 (25.9)	12.038^b^	0.017

Note: ^a^ One-way ANOVA, ^b^ chi-squared goodness-of-fit test

As shown in Table 4, there were significant differences in the genotype frequencies of rs53576 and rs237897 between the two groups (*p* < 0.05), while the statistical significances were lost after Bonferroni correction. However, the other six markers, namely, rs13316193 (*χ*^*2*^ = 1.581, *p* = 0.454), rs2254298 (*χ*^*2*^ = 1.231, *p* = 0.540), rs2268494 (*χ*^*2*^ = 3.016, *p* = 0.221), rs237889 (*χ*^*2*^ = 3.812, *p* = 0.149), rs1042778 (*χ*^*2*^ = 5.063, *p* = 0.080), and rs6770632 (*χ*^*2*^ = 1.068, *p* = 0.477), were not found to be statistically significant between the two groups.

**Table 4:** Genotype frequency differences between the BP and non-BP groups

Group	*n*	Genotype frequency			χ^2^	*p*
rs53576		AA	AG	GG		
BP group	329	184 (55.9)	20 (6.1)	125 (38.0)	6.054	0.048
Non-BP group	410	195 (47.6)	38 (9.3)	177 (43.2)		
rs237897		AA	AG	GG		
BP group	322	187 (58.1)	14 (4.3)	121 (37.6)	6.754	0.034
Non-BP group	404	205 (50.7)	34 (8.4)	165 (40.8)		

### Haplotype analyses

**Fig. 1:**
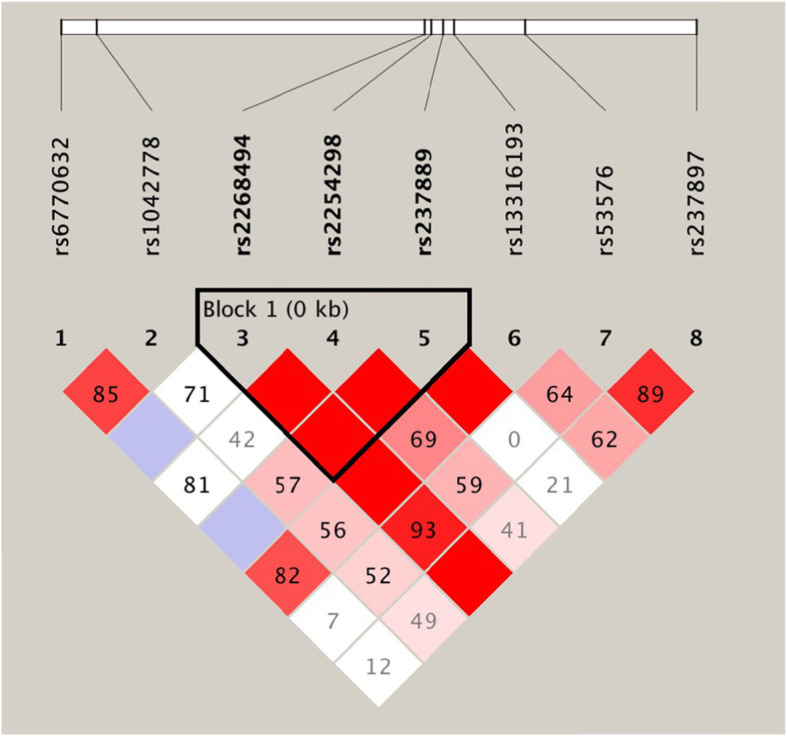
Linkage disequilibrium (LD) diagram of the eight SNPs in OXTR gene. The numbers in the squares represent D’ values, and the bright red squares represent that D’ = 1

Figure 1 presented the pairwise linkage disequilibrium (LD) results of the eight SNPs, and a haplotype block consisting of three SNPs (rs2268494, rs2254298, rs237897) was identified. There were no significant differences in the frequencies of these haplotypes between BP group and non-BP group (Table 5).

**Table 5:** Haplotype frequency differences between the BP and non-BP groups

Haplotype	BP group(*n* = 336)		Non-BP group(*n* = 421)		*p*	OR	95%CI
	*n*	f	*n*	f			
TGT	237.0	0.353	277.5	0.330	0.345	1.107	0.894–1.371
TAC	204.0	0.304	277.7	0.330	0.276	0.884	0.711–1.100
TGC	174.0	0.259	229.8	0.273	0.541	0.930	0.739–1.170
AGT	57.0	0.085	57.0	0.068	0.210	1.276	0.871–1.870

### Association analysis between childhood maltreatment and BPD

The impact of childhood maltreatment on BPD was examined by a linear regression analysis, in which BPD score was the dependent variable and total score of childhood maltreatment was the independent variable. The analysis revealed that childhood maltreatment was a significant positive predictor of BPD (*R*^*2*^ = 0.102, *β* = 0.322, *S. E* = 0.004 *t* = 9.384, *p* < 0.001). Because the total CTQ score had a significant independent effect on BPD, the five subtypes of childhood maltreatment were all entered into the whole model to determine whether any of the subtypes had an independent effect on BPD. The method of stepwise was used to control the multiple linear relationship between the independent variables. The results indicated that emotional abuse (*β =* 0.201*, S. E* = 0.021, *t* = 5.575, *p* < 0.001), and emotional neglect (*β =* 0.205*, S. E* = 0.014, *t* = 5.762, *p* < 0.001) could significantly predict BPD (*R*^*2*^ = 0.106, *F =* 46.444,*p* < 0.001), while physical abuse (*t* = 0.514, *p* = 0.607), sexual abuse (*t* = 0.542, *p* = 0.588) and physical neglect (*t* = 1.056, *p* = 0.291) were excluded from the multivariate linear regression model.

### Gene-environment interactions with respect to BPD

According to the results in 3.2, the two SNPs (rs53576 and rs237897) showed significant differences in genotype frequencies between the BP and non-BP groups, while these differences were not significant after Bonferroni correction. This indicates that the two SNPs may be related to the OXTR genetic variants, which further lead to the development of BPD. Therefore, we focus on the moderation roles of the two SNPs (rs237897 and rs53576) on the relationship between childhood maltreatment and BPD.

#### Interaction between OXTR rs53576 and childhood maltreatment

**Fig. 2:**
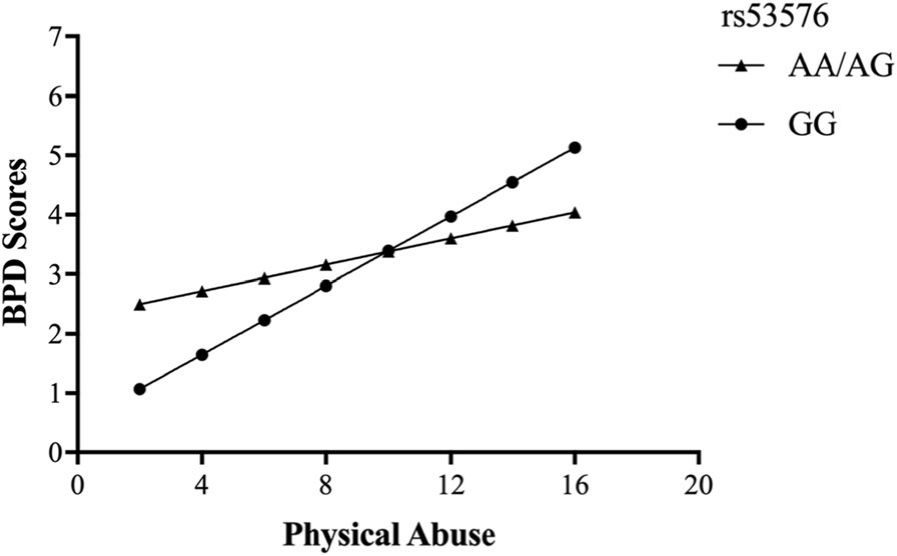
The interactive effect of OXTR rs53576 genotype and childhood physical abuse on BPD scores

**Fig. 3:**
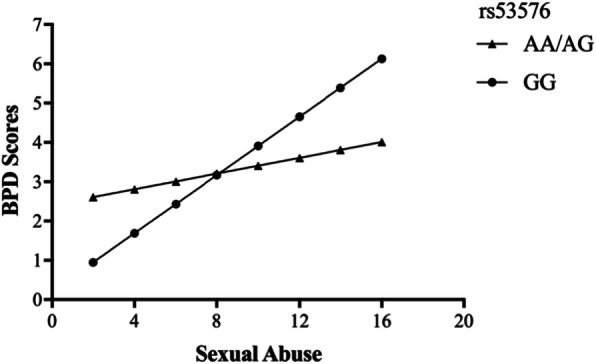
The interactive effect of OXTR rs53576 genotype and childhood sexual abuse on BPD scores

To examine the interaction between OXTR rs53576 and childhood maltreatment on BPD features in the high-risk inmates, we constructed a hierarchical linear regression. Genotype and maltreatment were entered on the first step, and the genotype × maltreatment interaction term was entered on the second step. The moderating role of OXTR rs53576 genotype on the relationships between physical abuse (*R*^*2*^ = 0.066, *t* = 2.723, *p* = 0.007), sexual abuse (*R*^*2*^ = 0.053, *t* = 2.914, *p* = 0.004) and BPD was significant, while childhood maltreatment total score (*R*^*2*^ = 0.105, *t* = 1.575, *p* = 0.116), emotional abuse (*R*^*2*^ = 0.077, *t* = 1.931, *p* = 0.054), emotional neglect (*R*^*2*^ = 0.071, *t* = − 1.016, *p* = 0.310) and physical neglect (*R*^*2*^ = 0.076, *t* = 0.990, *p* = 0.323) all failed to interact with genotype to predict BPD scores. As shown in Fig. 2, when subjects had lower physical abuse scores, those who carried the GG genotype had a lower risk of BPD than those who carried the A allele. While, when subjects had higher physical abuse scores, those who carried GG genotype had a higher risk of BPD than those who carried A allele. The interaction between OXTR rs53576 genotype and sexual abuse was similar. As shown in Fig. 3, at lower levels of sexual abuse, BPD scores were lower among those with GG genotype, while at higher levels of sexual abuse, BPD scores were lower among those with A allele.

The interactions between OXTR rs53576 and childhood abuse were also examined in the BP and non-BP groups. The results showed that neither childhood maltreatment total score (*R*^*2*^ = 0.033, *t* = − 0.216, *p* = 0.829; *R*^*2*^ = 0.040, *t* = 0.935, *p* = 0.351, respectively), nor emotional abuse (*R*^*2*^ = 0.021, *t* = 0.591, *p* = 0.555; *R*^*2*^ = 0.057, *t* = 1.318, *p* = 0.188, respectively), physical abuse (*R*^*2*^ = 0.015, *t* = − 0.311, *p* = 0.756; *R*^*2*^ = 0.043, *t* = 1.608, *p* = 0.109, respectively), sexual abuse (*R*^*2*^ = 0.003, *t* = − 0.339, *p* = 0.735; *R*^*2*^ = 0.030, *t* = 1.683, *p* = 0.093, respectively), emotional neglect (*R*^*2*^ = 0.035, *t* = 0.568, *p* = 0.570; *R*^*2*^ = 0.003, *t* = − 0.289, *p* = 0.773, respectively), physical neglect (*R*^*2*^ = 0.040, *t* = − 0.914, *p* = 0.361; *R*^*2*^ = 0.013, *t* = 0.572, *p* = 0.568, respectively) interacted with genotype to predict BPD scores in both group.

#### Interaction between OXTR rs237897 and childhood maltreatment

A two-way interaction model was conducted to examine the moderating effect of OXTR rs237897 on the relationship between childhood maltreatment and BPD features in the whole high-risk sample. The analysis revealed that the interactive effect of OXTR gene by childhood maltreatment total score was non-significant for BPD in high-risk inmates (*R*^*2*^ = 0.097, *t* = 0.341, *p* = 0.734). Similar non-significant results were obtained for emotional abuse (*R*^*2*^ = 0.070, *t* = − 0.402, *p* = 0.688), physical abuse (*R*^*2*^ = 0.056, *t* = 0.475, *p* = 0.635), sexual abuse (*R*^*2*^ = 0.040, *t* = 0.765, *p* = 0.444), emotional neglect (*R*^*2*^ = 0.066, *t* = 0.658, *p* = 0.511) and physical neglect (*R*^*2*^ = 0.072, *t* = 1.525, *p* = 0.128).

To examine the moderating effect of OXTR rs237897 on the relationship between childhood maltreatment and BPD features in the BP and non-BP groups respectively, we also constructed two-way interaction models. There were no significant interactions between genotype and childhood maltreatment total score (*R*^*2*^ = 0.029, *t* = 1.244, *p* = 0.214; *R*^*2*^ = 0.047, *t* = 1.608, *p* = 0.109, respectively), emotional abuse (*R*^*2*^ = 0.016, *t* = 0.772, *p* = 0.441; *R*^*2*^ = 0.060, *t* = 1.271, *p* = 0.204, respectively), physical abuse (*R*^*2*^ = 0.011, *t* = 0.471, *p* = 0.638; *R*^*2*^ = 0.044, *t* = 1.255, *p* = 0.210, respectively), sexual abuse (*R*^*2*^ = − 0.004, *t* = 1.114, *p* = 0.266; *R*^*2*^ = 0.030, *t* = 1.331, *p* = 0.184, respectively), emotional neglect (*R*^*2*^ = 0.032, *t* = 1.457, *p* = 0.146; *R*^*2*^ = 0.005, *t* = 0.199, *p* = 0.842, respectively), physical neglect (*R*^*2*^ = 0.034, *t* = 1.772, *p* = 0.077; *R*^*2*^ = 0.015, *t* = 1.550, *p* = 0.122, respectively) in both groups.

## Discussion

This study investigated genetic (OXTR) and environmental (childhood maltreatment) factors of BPD and highlighted G × E interactions relevant to BPD risk. In terms of genetic test results, the BP group had an increased rs53576 AA genotype frequency compared with the non-BP group. Although the difference did not survive after Bonferroni correction, this finding indicates that the high AA genotype frequency may increase the risk of BPD. This result is inconsistent with previous finding that the genotype groups for OXTR did not demonstrate differences in BPD scores [26]. However, these discrepancies may be due to the different analysis methods used to explore the relationship between rs53576 and BPD. Another possible explanation is ethnicity-related differences in allele frequency distribution. The frequency distribution of rs53576 alleles in this Asian population is opposite to that in Caucasian population, which indicates that the OXTR genetic variants in the two races are different. In the SNP rs237987, the higher AA genotype frequencies were found in BP group, while the difference was not significant after Bonferroni correction. The association between the specific oxytocin receptor marker rs237987 and BPD susceptibility has not previously been reported, so the results need to be verified in a larger and more representative sample.

With respect to the environmental aspect, the results showed that childhood maltreatment could positively predict BPD risk in the present study. This is not surprising given that evidence has accumulated suggesting that early childhood maltreatment is a risk factor for BPD. For instance, a retrospective study suggested that children who had been maltreated had an increased risk of presenting borderline personality features [51]. Another study in adult reported the association between experiencing traumatic events in childhood and an increased clinical severity of BPD in adulthood [52].

However, the present study only found that CTQ scores, emotional abuse and neglect could positively predict BPD risk. This may be due to the fact that a certain type of traumatic experience may increase the risk of specific mental illness in the trajectory of susceptible individuals from childhood to adulthood. For instance, a clinical study has found that emotional and sexual abuse may lead to BPD or BPD and attention deficit hyperactivity disorder (ADHD) comorbidity, while the history of physical abuse was related to the persistence of ADHD in adulthood [52]. From another perspective, the symptoms of BPD are heterogeneous, with multiple phenotypes, which may be caused by different factors in the development of individuals. A previous study suggested that affective instability was linked with childhood sexual abuse, while identity disturbance was associated with non-sexual developmental trauma among BPD patients [53].

The present study found OXTR gene×childhood maltreatment interactions only in the high-risk inmates, while no significant interaction was found in BP group and non-BP group. This indicates there are no significant gene×maltreatment×group three-way interactions, and the pattern of interactions in this study are only applicable to high-risk prison inmates. In the OXTR rs53576, the GG carriers were strongly affected whether the physical abuse or sexual abuse was low or high, which conforms to the hypothesis of gene plasticity [3]. These results are consistent with the prior literature showing that GG carriers may be more sensitive to social cues and more prosocial than A-allele carriers, while this attunement for social cues may be a disadvantage under adverse circumstances such as early childhood maltreatment [54]. In contrast, a recent study reported that A-allele carriers had higher levels of BPD symptoms under negative family conditions and lower levels under positive conditions, GG genotype carriers had average levels of BPD symptoms regardless of their family quality [26]. Considering there are ethnic-related differences in the genotype frequency of rs53576, genetic susceptibility may also vary among different races. However, previous studies in Caucasian [32], African American [55] and multi-ethnic groups [56] found that carriers of at least one rs53576 G-allele were more susceptible to negative life experiences. In addition, a meta-analysis suggested that GG homozygotes had higher general sociality than A-allele carriers both in Caucasians and Asians [57]. These results indicate that GG genotype carriers of OXTR rs53576 might be more sensitivity to environment, which was not affected by ethnicity. This may partially explain the findings of the present study.

Another possibility is that the contradictory results of genetic susceptibility might be attributable to gender differences. A study in low-income children of different ethnicities found that girls who carried the A allele were more susceptible than those with GG genotype, while boys with the GG genotype were more susceptible than those with A allele [33]. A recent study confirmed that the A allele carriers were more susceptible to childhood maltreatment in females [58]. Thus, it may mean there are different patterns of OXTR gene and childhood maltreatment interactions in different genders. Future research needs to clarify the exact mechanism.

It is noted that the present study found the total CTQ scores, emotional abuse, emotional neglect and physical neglect did not show significant interactions, which could significantly predict BPD scores. In contrast, physical and sexual abuse interacted with genotype to predict BPD scores, while these two types of abuse did not significantly predict BPD scores. These findings indicate that total CTQ scores, emotional abuse and emotional neglect have independent predictive effects on BPD, while the effects of physical and sexual abuse on BPD depend on the genetic characteristics of individuals. Another plausible explanation is that OXTR rs53576 interacts with physical and sexual abuse to influence OXTR transcription and expression. There is evidence that physical exposure, including physical abuse and sexual abuse, showed strongest association with methylated variation [59].

The explanation for the significant interactions between gene and environment should be cautious. The prior literature reported that adolescents whose mothers had BPD experienced more maltreatment compared with those whose mothers were normal [60], which revealed that the intergenerational transmission of BPD may be through childhood maltreatment. Another study showed that mothers’ maltreatment history was a more strongly prediction for mother-infant attachment disorganization score among mothers with more plasticity alleles of OXTR gene [61], which suggested that maternal maltreatment history would influence offspring depending on the maternal genetic characteristics. Hence, both genetic and environmental factors are contributed to the development of BPD. Whether adverse environment lead to changes in biomarkers or whether genetic basis increases susceptibility to adverse environment remains future research to explore.

There are several strengths in the study. First, eight OXTR SNPs were genotyped based on the previous literature, which is helpful to understand the effect of OXTR genetic variants on BPD. In addition, to our knowledge, this is the first study to demonstrate interactive effects between OXTR polymorphisms and childhood maltreatment subtypes on BPD in Chinese male high-risk inmates. Given the characteristics and high risk for BPD, this population is worth investigating. The findings that the patterns of G × E interactions in prison sample contribute both to prison management and to treatment or even prevention of BPD.

This study also has several limitations that need to be acknowledged. The current sample consists of male high-risk inmates, a population that is at risk for BPD. Thus, it is possible that the results would differ in the general population. Given that the different effects of oxytocin in different genders [33], the findings also cannot be generalized to females. Participants were only assessed with self-reported questionnaires. Although the BPD and ASPD subscales in PDQ-4 as well as CTQ-SF had a good reliability in this study, self-reporting may lead to potential bias due to their own memory of events. In addition, the subjects who had higher levels of BPD were likely to exaggerate the adverse events they experienced in early life, which would inflate the association between childhood maltreatment and BPD. The onset and duration of childhood maltreatment were not considered, so the interpretation and application of the results should be cautious.

## Conclusion

In summary, the findings of the present study indicated that OXTR gene variations and childhood maltreatment are both risk factors for the development of BPD and that OXTR polymorphisms interact with childhood maltreatment subtypes to predict BPD scores. These interaction findings reinforced the view that OXTR is a plasticity gene that is associated with increased sensitivity to the environment, regardless of negative or positive [56, 62]. The results may contribute to understand the pathological mechanism of BPD, further guiding the early identification of personality disorders and the selection of interventions.

## Supplementary information

**Additional file 1.** Table S1. Primers used in the screening of SNPs

## Data Availability

The datasets used and analyzed during the current study are available from the corresponding author on reasonable request.

## Abbreviations

BPD: Borderline personality disorder
OXTR: Oxytocin receptor
ASPD: Antisocial personality disorder
HTR: Serotonin receptor
TPH: Tryptophan hydroxylase
MAOA: Monoamine oxidase A
COMT: Catechol-O-methyltransferase
DAT: Dopamine transporter
SNP: Single nucleotide polymorphism
ASD: Autism Spectrum Disorder
CU: Callous-unemotional
G × E: Interaction between the gene and the environment
PDQ-4: Personality Diagnostic Questionnaire-4
BIS-11: Barratt Impulsiveness Scale-11
CTQ-SF: Childhood Trauma Questionnaire Short Form
CHB: Han Chinese in Beijing, China
SPSS: Statistical Product and Service Solutions
HWE: Hardy-Weinberg equilibrium
LD: Linkage disequilibrium
ANOVA: Analysis of variance
MAF: Minor allele frequencies
ADHD: attention deficit hyperactivity disorder
