# Supplementary material for: Oxytocin receptor gene, childhood maltreatment and borderline personality disorder features among male inmates in China
Source: BMC Psychiatry. 2020 Jun 24;20:332. doi: 10.1186/s12888-020-02710-0 (PMC7315490; doi:10.1186/s12888-020-02710-0)
Supplement: Supplementary file 1 — Additional file 1. Table S1. Primers used in the screening of SNPs [file 12888_2020_2710_MOESM1_ESM.docx]

**Table S1.** **Primers used in the screening of SNPs**

| **SNPs** |  | **Primers** |
| --- | --- | --- |
| rs237897 | Forward | CTTTCTTCTCTTTCCTCCAAGTG |
|  | Reverse | AACCAAGCACCTAGTAGTTGAAG |
| rs13316193 | Forward | TAGTTTAAAAAGCAGAACAAGGGG |
|  | Reverse | ACATTTTAGGCTGAACAGTCTTTG |
| rs237889 | Forward | CACCCTGTTCATAGACCATGAGG |
|  | Reverse | GATGATTTGCCGCTTTCCAC |
| rs2254298 | Forward | ACCTTGACCACACGGTCCCAC |
|  | Reverse | GCCCCTTTCAGGAAACCATCC |
| rs2268494 | Forward | ACCTTGACCACACGGTCCCAC |
|  | Reverse | GCCCCTTTCAGGAAACCATCC |
| rs1042778 | Forward | ATACTGGAGTGAAATTACAAGTCC |
|  | Reverse | CTAGGTGATGGCGTATGTTTGTG |
| rs53576 | Forward | TGAATATCCTGTCCAAGCTTCTC |
|  | Reverse | AACTGTTTCCCCATCTGTAGAATG |
| rs6770632 | Forward | ttcataaggattgcactgaatctg |
|  | Reverse | gcaacaaacaatatgaaaacgaag |
